# Rapid Recuperation After Surgical Intervention and Rehabilitation in a Patient With Spinal Hemangioma: A Case Report

**DOI:** 10.7759/cureus.55114

**Published:** 2024-02-28

**Authors:** Rishika Gabada, Pallavi Harjpal

**Affiliations:** 1 Department of Neuro-Physiotherapy, Ravi Nair Physiotherapy College, Datta Meghe Institute of Higher Education and Research, Wardha, IND

**Keywords:** decompressive laminectomy, physiotherapy, rehabilitation, surgical intervention, spinal hemangioma

## Abstract

This case report describes the remarkable recovery journey of a 42-year-old male who suffered from neurological symptoms over three months, including tingling in both lower extremities. It was determined that a spinal hemangioma, a normally non-cancerous medical ailment, was the cause of these symptoms, which were also accompanied by difficulty walking and problems with bowel and bladder incontinence. A laminectomy and spinal cord decompression surgery were the two most significant medical procedures the patient underwent as part of his treatment, followed by a carefully structured rehabilitation program, as part of a holistic approach. Astoundingly, the patient's physical condition showed considerable improvements in several areas just one week after surgery. The reduction of pain, increased range of motion (ROM), and increased muscular strength were the aspects where these changes were most noticeable. This quick recovery reflects the benefit of combining surgical and rehabilitation techniques in these patients. The patient was prescribed a home exercise program (HEP) at the time of his discharge from the hospital so that he could continue his recovery independently in the comfort of his own home. This HEP was created to ensure that the patient could keep up and continue to make progress.

This case report sheds light on the benefits of adopting a comprehensive strategy while treating spinal hemangiomas. The combined efforts of the surgical and rehabilitation therapy teams greatly improved the patient's prognosis. This aspect of synergy helped develop a whole treatment strategy that included both surgical tumor removal and crucial postoperative rehab for optimum healing and function.

## Introduction

Spinal hemangiomas are the most typical primary tumors detected in the spine [1]. With a documented prevalence rate of 26%, these disorders typically have no symptoms and start in the vertebral body [2]. Vertebral hemangiomas (VHs) were first identified by Virchow in 1867, and Perman was the first to identify them radiologically in 1926 [3]. While VHs most frequently affect the thoracic spine, in up to 30% of cases, these lesions can affect multiple levels [4]. VHs, also known as non-cancerous lesions, result from aberrant embryonic development. They are made up of benign vascular growths produced by endothelial cells that appear as tiny blood vessels penetrating the central cavity of the vertebrae and the bone marrow. These lesions often only affect the vertebral bodies [5]. These conditions are frequently asymptomatic and can either be found by chance or remain completely undiagnosed [6].

Hemangiomas are common non-cancerous growths in the spine; autopsy studies have found that their incidence is around 11% [7]. Hemangiomas can occasionally become active and are referred to as aggressive or compressive vascular malformations. When these anomalies become symptomatic, they may cause pain and myelopathy due to issues like bleeding inside the spinal cord, bone tissue growth, or extension into nearby soft tissues and the neural structures at the back [8]. These tumors are usually seen on body and spine scans performed with CT and MRI, leading to the typical accidental detection of these growths. They are frequently observed on X-rays of the thoracolumbar spine as well. VHs frequently exhibit the recognizable "corduroy cloth" or "jail bar" pattern [9]. Females are generally more likely than males to be affected by this condition [10]. Only 0.9-1.2% of these anomalies lead to clinical presentations, and symptoms constitute back pain and neurological problems in the majority of patients [11].

A complete transverse lesion of the spinal cord may develop from hemangiomas when accompanied by considerable spinal column growth or epidural penetration, and this constitutes a typical clinical presentation [12]. The symptoms of hemangiomas in the vertebrae can range from minor localized pain and discomfort to more serious neurological problems [13]. Back pain symptoms can range widely in intensity; some people may have severe pain that worsens with movement, while others may only experience mild to moderate discomfort. An individual's quality of life and ability to carry out everyday activities can be significantly affected by back pain, which is a common and complex condition [14]. The production of hematomas from vascular lesions, cord compression, epidural extension with neurologic impairments, and nerve root involvement resulting in neurological symptoms are all rare sequelae of pathologic burst fractures [15]. Hemangiomas frequently extend into the spinal canal or neural apertures in cases of neurogenic pain [16]. The option of embolization followed by a decompressive laminectomy is offered to patients with a neurological impairment caused by a lesion limited to either the vertebral body or the posterior components [17].

## Case presentation

A 42-year-old male patient was admitted to the neurosurgery department with chief complaints of tingling sensation in bilateral lower limbs for three months and difficulty in walking for two months with bowel and bladder incontinence. The contrast MRI, performed the day after admission, showed heterogeneously enhancing altered marrow signal noted in the D5 vertebral body appearing hyperintense on T1- and T2-weighted and short tau inversion recovery (STIR) images. There was an enhancing epidural soft tissue component noted at the D5 level causing moderate narrowing of the central canal with compression of the dorsal cord. The altered marrow signal was seen extending posterior elements of the D5 vertebra. Intramedullary, T2 hyperintense signal was noted in the dorsal at the D5 level. The spinal canal anteroposterior (AP) diameter at the D5 level was 5.2mm. Hemangiomas were noted in the C7 and D4 vertebral bodies. There was no history of addictions at the time of the presentation. The patient was admitted to the hospital on September 17, 2023. His surgery was performed on September 20 and then physiotherapy was started on September 22.

Clinical findings

**Figure 1:**
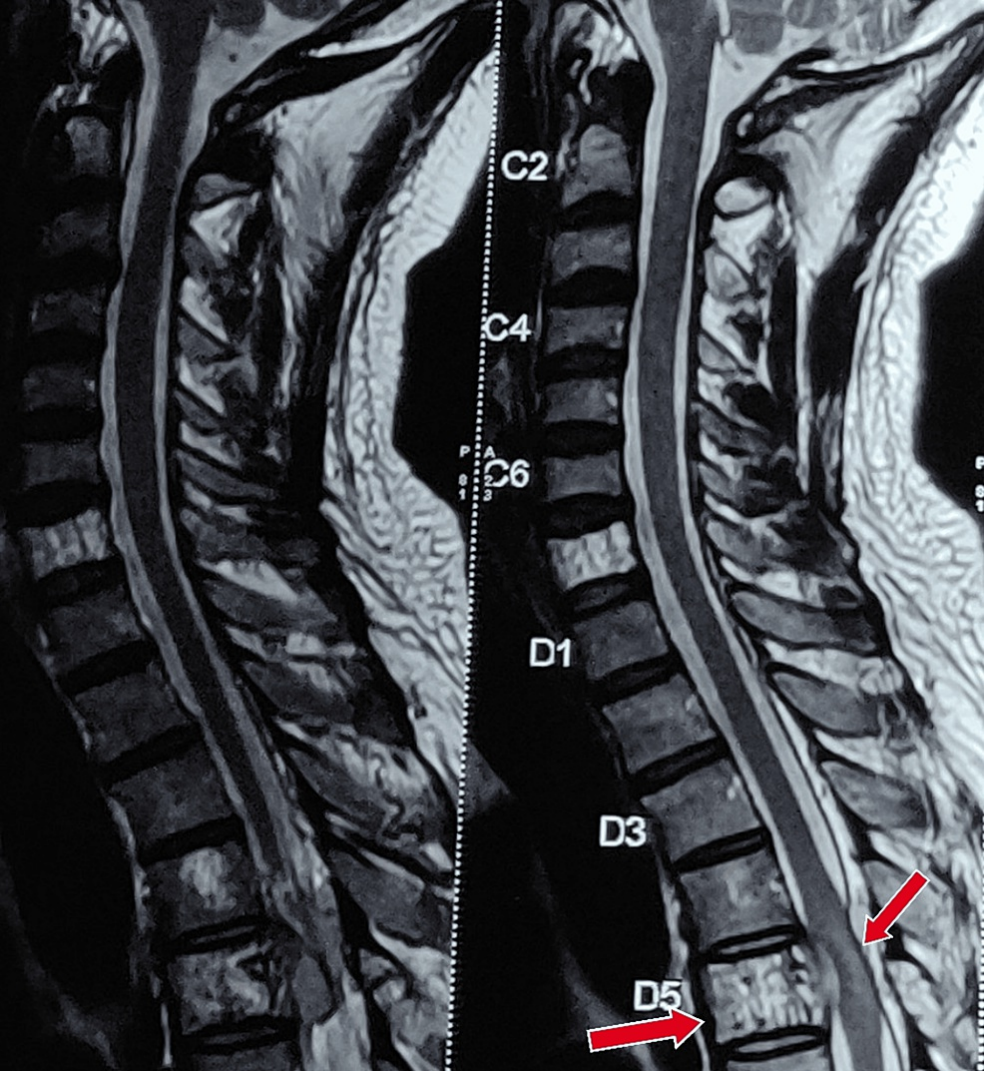
The MRI shows heterogeneously enhancing altered marrow signal noted in the D5 vertebral body There is an enhancing epidural soft tissue component noted at the D5 level causing moderate narrowing of the central canal with compression of the dorsal cord MRI: magnetic resonance imaging

Before the examination, the patient's consent was obtained. The examination findings on the second postoperative day are presented in Table 1. Figure 1 shows an MRI of the D5 vertebra. On the numerical pain rating scale (NPRS), the score was 6/10 on rest and 9/10 on movement.

**Table 1:** Clinical findings on postoperative day two

Variable	Movement	Postop day 2	
Range of motion	Active range of motion		
Shoulder joint	Flexion	0^o^-70^o ^	
	Extension	0^o^-20^o ^	
	Abduction	0^o^-90^o^	
Hip Joint	Flexion	0^o^-50^o^	
	Extension	0^o^-10^o^	
Manual muscle testing	Muscle group	Right	Left
	Shoulder flexors	2/5	2/5
	Shoulder extensors	2/5	2/5
	Elbow flexors	3/5	3/5
	Elbow extensors	3/5	3/5
	Wrist flexors	4/5	4/5
	Wrist extensors	4/5	4/5
	Hip flexors	2/5	2/5
	Knee flexors	3/5	3/5
	Knee extensors	3/5	3/5
	Ankle plantar flexors	3/5	3/5
	Ankle dorsi flexors	3/5	3/5

Medical management

The patient underwent D4 and D5 decompressive laminectomy with the removal of extradural mass with (hemangiomatous) decompression of the cord with bilateral D4 and D6 perpendicular screws and rod fixation. Medications administered were as follows: tablet gabapentin NT 100 mg once a day, cap Felicita once a day, tablet pan 40 mg twice a day, and tab Dexa 4 mg thrice a day.

Physiotherapy management

**Figure 2:**
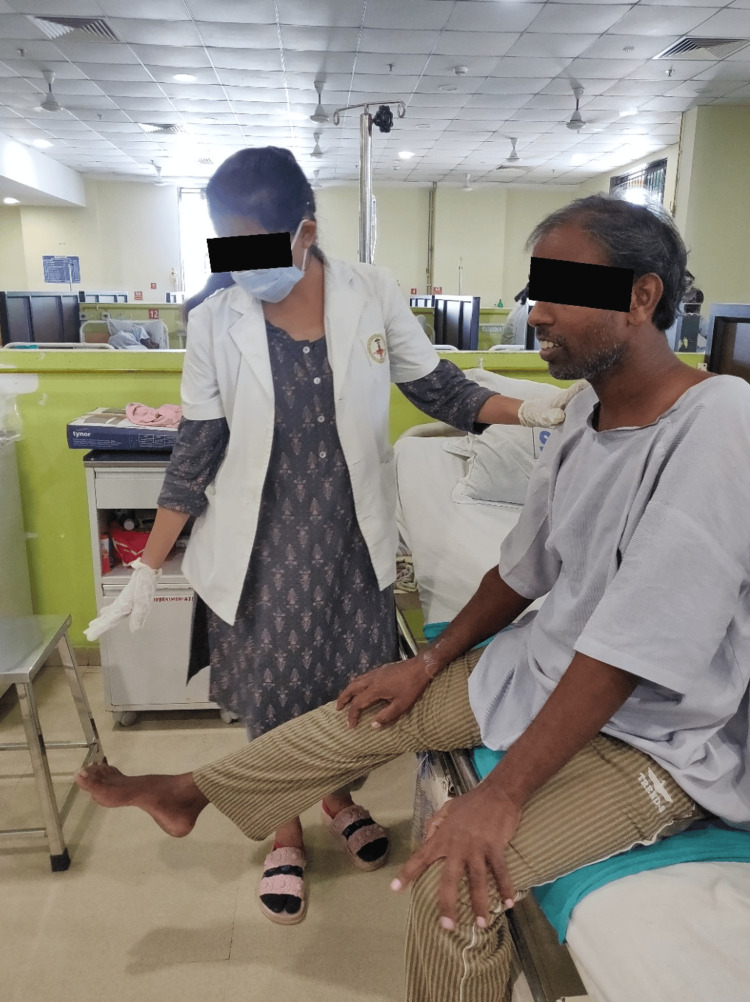
Patient performing dynamic quadriceps

**Figure 3:**
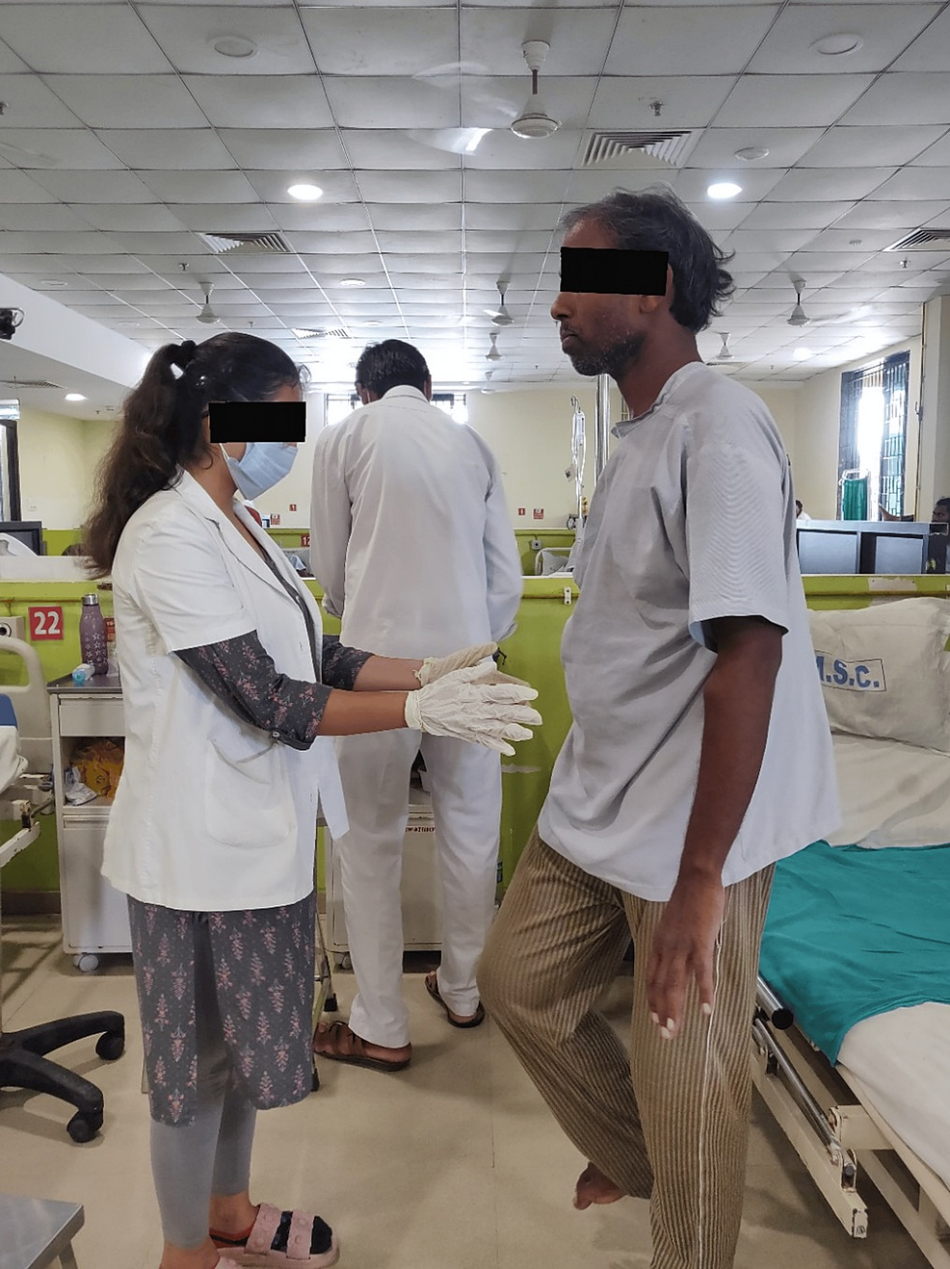
Patient performing spot marching without assistance

Table 2 describes the physiotherapy protocol from postoperative day two to postoperative day eight. Figures 2-3 show the rehabilitation course.

**Table 2:** Physiotherapy treatment protocol, week one (days two to eight) PNF: proprioceptive neuromuscular facilitation

Goal	Intervention	Duration
To improve range of motion	Active range of motion for shoulder joint, elbow joint, and hip joint in all planes [18]	10 repetitions, 3 times per day
To improve strength	Isometric strengthening exercises for hip and knee joints, and upper-limb strengthening using a 1/2 kg weight cuff [18]	10 repetitions, 3 times per day
To improve pelvic stability and weakness	Pelvic PNF patterns	10 repetitions, 3 times per day
To improve trunk control	Pelvic PNF and bed mobility training	10 repetitions, 3 times per day
Balance training	Sitting and standing training	2 times/day
Functional training	Bed mobility training	15 repetitions, 3 times per day

Home exercise program

As part of the home exercise program (HEP), the patient has been instructed to continue performing all the exercises they learned during the inpatient stay. This continuity is essential to maintain and build upon the progress achieved while under observation. By incorporating these exercises into their daily routine at home, the patient can continue to strengthen and rehabilitate the affected areas, as well as prevent the recurrence of any underlying issues. Table 3 illustrates the HEP protocol.

**Table 3:** Home exercise program ROM: range of motion; PNF: proprioceptive neuromuscular facilitation

Category	Exercises and activities	Duration	Frequency
Enhance ROM	Shoulder joint: active ROM exercises in all directions	10 repetitions for each joint	Three times per day
	Elbow joint: active ROM exercises		
	Hip joint: active ROM exercises in various planes		
Strengthen muscles	Hip and knee Joints: isometric strengthening exercises	10 repetitions of each exercise	Three times per day
	Upper limb: 1/2 kg weight cuff for strengthening exercises		
Enhance pelvic stability	Pelvic PNF patterns: execute PNF patterns	10 repetitions	Three times per day
Improve trunk control	Balance training: various balance exercises	15 repetitions of each exercise	Three times per day
	Functional training: incorporate functional movements		
Additional exercises	Core strengthening: planks, leg raises, and Russian twists	10 repetitions of each exercise	Three times per day
Stretching	Stretching: targeting major muscle groups	Hold each stretch for 20-30 seconds	Three times per day
Cardiovascular conditioning	Aerobic exercises: Brisk walking, cycling	At least 30 minutes daily	-
Joint mobility	Joint mobility exercises for wrists, ankles, neck	10 repetitions for each joint	Three times per day
Breathing exercises	Deep breathing exercises for lung capacity	Perform for 5-10 minutes	Three times per day

Follow-up and outcomes

The details of the patient's evaluation on postoperative day eight are shown in Table 4. Outcome measures post-rehabilitation are summarized in Table 5.

**Table 4:** Evaluation on postoperative day eight and outcomes

Variable	Movement	Postop day 2		Postop day 8	
Range of motion					
Shoulder joint	Flexion	0^0^-70^0^		0^0^-150^0^	
	Extension	0^0^-20^0^		0^0^-50^0^	
	Abduction	0^0^-90^0^		0^0^-150^0^	
Hip joint					
	Flexion	0^0^-50^0^		0^0^-100^0^	
	Extension	0^0^-10^0^		0^0^-20^0^	
Manual muscle testing	Muscle group	Right	Left	Right	Left
	Shoulder flexors	2/5	2/5	3/5	3/5
	Shoulder extensors	2/5	2/5	3/5	3/5
	Elbow flexors	3/5	3/5	4/5	4/5
	Elbow extensors	3/5	3/5	4/5	4/5
	Wrist flexors	4/5	4/5	4/5	4/5
	Wrist extensors	4/5	4/5	4/5	4/5
	Hip flexors	2/5	2/5	3/5	3/5
	Knee flexors	3/5	3/5	4/5	4/5
	Knee extensors	3/5	3/5	4/5	4/5
	Ankle plantar flexors	3/5	3/5	4/5	4/5
	Ankle dorsi flexors	3/5	3/5	4/5	4/5

**Table 5:** Outcome measures

Outcome measures	Pre-rehabilitation	Post-rehabilitation
Numerical pain rating scale	8/10	4/10
Berg Balance Scale	22/56	49/56
Dynamic gait index	10/24	19/24

## Discussion

Hemangiomas are usually benign growths that often go unnoticed. The lesions, however, can become troublesome when the neural arch widens as they may directly compress the thecal sac, and nerve roots, or enlarge the vertebral body, which would result in symptoms [18]. In this comprehensive case study, we engage in an in-depth assessment of the rehabilitation of a 42-year-old patient who underwent a challenging surgical procedure. The surgical procedure included extradural mass removal, D4 and D5 decompressive laminectomy, cord decompression, and bilateral D4 and D6 perpendicular screw and rod fixation. The patient's recovery and postoperative care were of utmost importance due to the complicated nature of the surgery. The patient showed considerable improvement in their condition during the first week after the operation itself. This quick recuperation was credited to a properly planned rehabilitation program that was created to handle the particular difficulties raised by the surgery. The patient's functional recovery and general well-being were optimized by a variety of therapies included in the rehabilitation process. The establishment of a HEP was a crucial component of the rehabilitation strategy. The patient's continuing recovery process is supported and sustained by the meticulously constructed HEP. It included a series of activities and exercises tailored to the patient's unique requirements and capabilities. The HEP made sure that rehabilitation efforts continued smoothly after the patient was discharged from the hospital by acting as a key link between the hospital and the patient's care at home.

A pertinent study has been carried out by Brindisino et al., which draws on the larger landscape of rehabilitation research. Their eight-week rehabilitation program offers helpful insights into organized and all-encompassing rehabilitation strategies. The program starts with an important phase of patient education, ensuring that people are aware of the importance of their rehabilitation journey and are actively involved in it. Basic workouts that establish the groundwork for future advancement follow this initial phase. Myofascial therapy, muscle-strengthening exercises, and mobilization treatments are gradually introduced as the rehabilitation program progresses. The patient's physical strength and mobility are being gradually rebuilt through this step-by-step process [19]. In another study, carried out by Chu and Leung, the patient underwent conservative treatment, including robotic traction therapy and thoracic spinal adjustments, to address intervertebral limitations while maintaining the hemangioma site pristine [20].

## Conclusions

This case report highlights the value of treating patients with spinal hemangiomas holistically in terms of care and rehabilitation. Our patient's pain levels were found to be reduced when measured with NPRS, ROM increased as measured by a goniometer, and muscular strength also increased according to the MRC grading system. Also, static and dynamic balance improved based on the Berg Balance Scale and dynamic gait index. The surgery, when combined with a well-designed physiotherapy program and HEP, leads to an overall improvement in the patient’s condition. This report emphasizes the great potential for favorable patient outcomes when a multidisciplinary strategy is used in the treatment of spinal hemangiomas, stressing the importance of both surgical and rehabilitation therapies.
